# Ethnopharmacology, phytochemistry, and pharmacology of *Rubus parvifolius* L (Máoméi): evidence and translational challenges

**DOI:** 10.3389/fphar.2026.1871424

**Published:** 2026-08-03

**Authors:** Boshi Duan, Chi Zhang, Xiao Hu, Xue Bai, Hu Peng, Han Zhao

**Affiliations:** 1 State Key Laboratory of Bioactive Substance and Function of Natural Medicines, Institute of Materia Medica, Chinese Academy of Medical Sciences & Peking Union Medical College, Beijing, China; 2 Respiratory Medicine Department, The University of Hong Kong-Shenzhen Hospital, Shenzhen, Guangdong, China; 3 Department of Medical Oncology, National Cancer Center/National Clinical Research Center for Cancer/Cancer Hospital & Shenzhen Hospital, Chinese Academy of Medical Sciences and Peking Union Medical College, Shenzhen, China

**Keywords:** ethnopharmacology, máoméi, nigaichigoside F1, phytochemistry, Rubus parvifolius, suavissimoside R1, triterpenoid saponins, ursolic acid

## Abstract

**Background:**

*Rubus parvifolius* L (Máoméi; Rosaceae) is a medicinal shrub widely used in southern Chinese folk medicine for haemorrhage, traumatic injury, inflammatory disorders, and certain cancers. Over the past 3 decades, increasing phytochemical and pharmacological investigation has expanded the preclinical literature for this species.

**Objective:**

This review critically summarises the ethnomedicinal background, phytochemistry, pharmacology, extraction methodology, quality control, and toxicological evidence relating to *R. parvifolius*, while identifying key translational limitations and future research priorities.

**Methods:**

Literature published between 1988 and May 2026 was retrieved from PubMed, Web of Science, CNKI, and SciFinder using “*Rubus parvifolius*” and its major Chinese synonyms (茅莓, 蛇泡簕, 三月泡) without language restriction.

**Results:**

More than 60 individual metabolites have been identified across seven structural classes, with ursane- and oleanane-type pentacyclic triterpenoids and their saponins as the predominant pharmacologically investigated class. Preclinical studies—primarily of TSRP (total saponins of *R. parvifolius*) — report anti-tumour, neuroprotective, hepatoprotective, anti-inflammatory, antibacterial, antioxidant, anti-fatigue, and anti-osteoclastogenic activities in cell-based and rodent models. Seven studies published during 2024–2025 have added substantially to this evidence base, contributing a four-tier botanical identification framework, an integrated UPLC-MS/MS plus network pharmacology characterisation of root metabolites, and the first reports of imatinib-resistance reversal and of ferroptosis-associated hepatic activity. A recent quantitative characterisation of leaf-flavonoid composition in an atopic dermatitis model and a GC-MS chemotaxonomic comparison across three *Rubus* species further refine the species-specific evidence base. The evidence remains predominantly preclinical, with mechanistic findings consisting largely of pathway-associated expression changes rather than experimentally validated mechanisms.

**Conclusion:**

*R. parvifolius* possesses a pharmacologically diverse chemical profile with broad preclinical activity consistent with several traditional indications. Current evidence is preclinical and should be regarded as hypothesis-generating. Future priorities include pharmacokinetic studies, standardised extract characterisation, regulatory-grade toxicology, and clinically relevant oral-route validation.

## 1 Introduction

*Rubus parvifolius* L [Rosaceae; *Rubi* parvifolii radix et rhizoma—root and rhizome as the principal medicinal drug in regional pharmacopoeias] (Subfamily *Rosoideae*), commonly known as Máoméi in Chinese—and historically also as Shé Pāo Lè, Sān Yuè Pāo, and Hóng Méi Xiāo in regional sources—is a deciduous prickly shrub widely distributed across central, eastern, and southern China, Japan, Korea, Taiwan, Vietnam, and parts of Southeast Asia. The species has been used in regional Chinese ethnomedicine, in southern provinces, for haemorrhagic conditions, traumatic injuries, hepatitis, nasopharyngeal carcinoma, nephritis, leukaemia, rheumatic pain, and dysentery. In Guangdong province, *Rubus parvifolius* (under the local name Shé Pāo Lè) is classified as a designated ‘local medicinal botanical drug’ with a quality monograph in the 2019 Guangdong Provincial Standard for Chinese Medicinal Materials.

Phytochemical investigation of *R. parvifolius* began in 1988 with the isolation of (−)-epicatechin from the fresh roots (Section 5.4), and pharmacological investigation followed in 1990, when Zhu et al. (1990) reported haemostatic-fibrinolytic, cardiotonic, and anti-hypoxic activities of the aqueous root extract. Choi et al. (1991) reported the first triterpenoid metabolites in 1991. The chemical foundation established by these early studies has subsequently been extended to over 60 identified metabolites across seven structural classes (Section 5). The past 3 decades have produced increasing preclinical investigation, with reported activities spanning anti-tumour, neuroprotective, hepatoprotective, antibacterial, anti-inflammatory, anti-fatigue, and anti-bone-resorption endpoints across multiple *in vitro* systems and rodent models (Tan et al., 2003a; Du and Rao, 2003; Du et al., 2005; Wang Y. et al., 2006; Liang et al., 2011; Cai et al., 2012; Zou and Huang, 2012; Zhang et al., 2016a; Zhang et al., 2016b; Hu, 2011; Wei et al., 1996; Lu et al., 2024; Jia et al., 2025; Lin et al., 2021; Huang et al., 2016). Studies published during 2024–2025 report ferroptosis-associated signalling in liver fibrosis (Wang et al., 2025), PI3K/AKT-associated signalling in imatinib-resistance models (Cheng et al., 2025), and an integrated UPLC-MS/MS plus network pharmacology characterisation of root metabolites (Jia et al., 2025).

Despite the growing literature, no comprehensive English-language critical review focused specifically on *R. parvifolius* has integrated the recent 2024–2025 mechanistic literature. Earlier Chinese-language sources, including the historical herbal-textual research by Chen and Mei (2015) and a recent 2024 review of pharmacological effects by Zhang et al. (2024), have provided valuable but partial syntheses, and have not incorporated the 2024–2025 mechanistic literature. The present review addresses this gap.

Two framing considerations apply throughout. First, the pharmacological evidence base consists almost entirely of cell-based and small-animal model studies; clinical data are very limited and no randomised controlled trial of any *R. parvifolius* preparation has been published. Findings should be interpreted as preclinical and hypothesis-generating. Second, publication bias is likely to operate in this literature, as negative and null findings in preclinical studies of traditional medicines are systematically under-reported; the positive findings summarised here may therefore overestimate the true effect size and consistency of activities. These caveats are revisited in Sections 7, 10.

## 2 Methods

**FIGURE 1:**
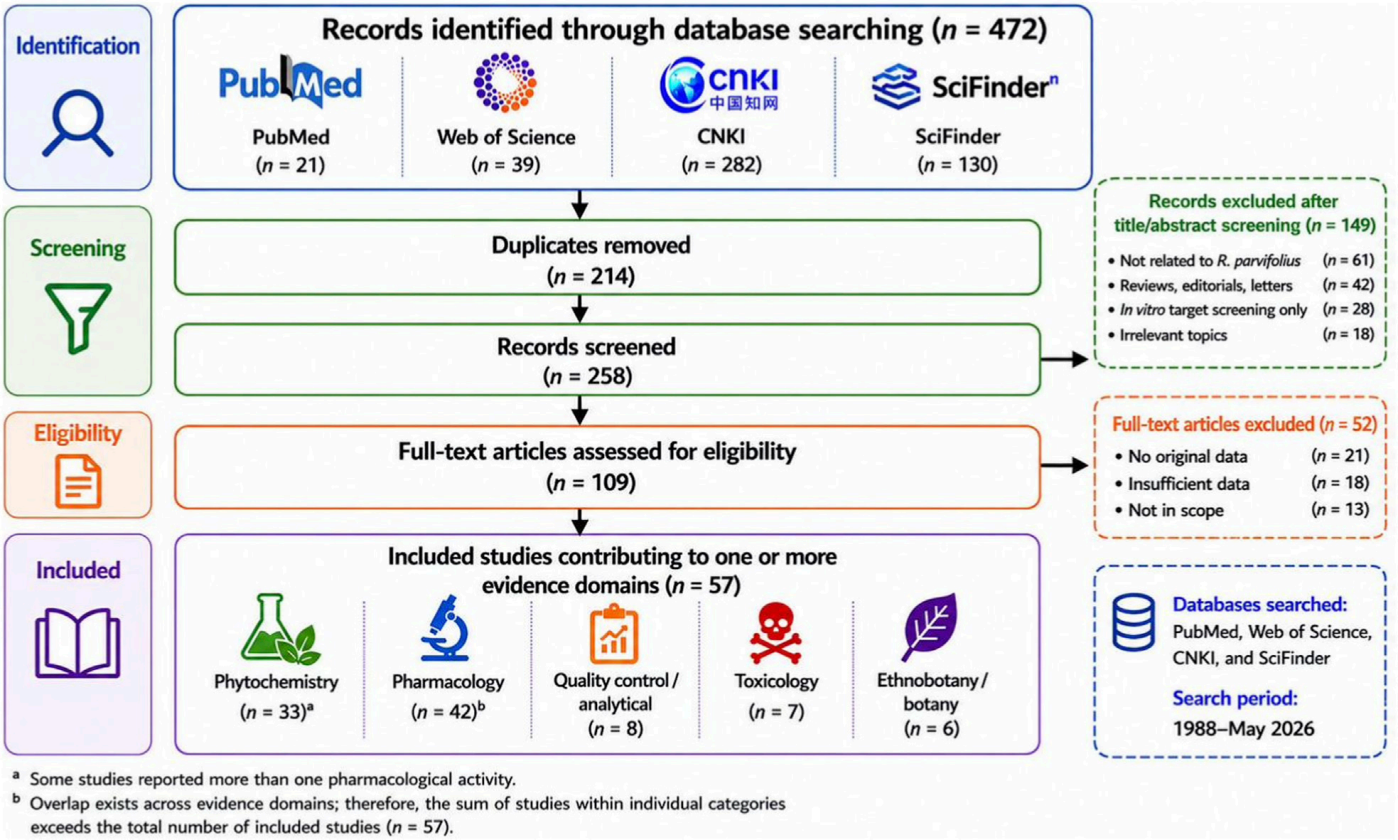
Study-selection workflow. 472 records were identified across PubMed (n = 21), Web of Science (n = 39), CNKI (n = 282), and SciFinder (n = 130); 214 duplicates were removed; 258 records were screened by title and abstract, with 149 excluded (61 not related to *Rubus parvifolius*; 42 reviews/editorials/letters; 28 *in vitro* target screening only; 18 irrelevant topics); 109 full-text articles were assessed for eligibility, with 52 excluded (21 no original data; 18 insufficient data; 13 not in scope); 57 studies were included in the final synthesis. Included studies contribute to one or more evidence domains: phytochemistry (n = 33), pharmacology (n = 42), quality control/analytical (n = 8), toxicology (n = 7), ethnobotany/botany (n = 6); overlap exists across domains, therefore the sum exceeds the total of 57 unique studies.

This narrative review follows the PRISMA 2020 (Preferred Reporting Items for Systematic Reviews and Meta-Analyses) approach for transparent reporting of literature identification, screening, eligibility, and inclusion, with numerical accounting shown in Figure 1. No formal risk-of-bias assessment or quantitative meta-analysis was performed, given the substantial heterogeneity in extract type, model, and dosing across the primary literature.

### 2.1 Search strategy

Literature relating to *R. parvifolius* was retrieved from four bibliographic databases—PubMed, Web of Science, CNKI (China National Knowledge Infrastructure), and SciFinder—covering the period 1988 to May 2026 (the final search date) with no language restriction. The exact search strings were: for English databases, “*R. parvifolius*” OR “*R. parvifolius* L.”; for CNKI, 茅莓 OR 蛇泡簕 OR 三月泡 OR 红梅消 OR “*R. parvifolius*” (combined). Additional sources were identified through forward and backward citation tracing of key reviews and high-cited primary studies, and through consultation of authoritative regional materia medica, including the Pharmacopoeia of the People’s Republic of China (2020 edition), the Guangdong Provincial Standard for Chinese Medicinal Materials (Third Volume, 2019, pp. 312–315) (Guangdong Food and Drug Administration (compiled), 2019), and the Bencao Shiyi (Tang dynasty).

### 2.2 Inclusion criteria

Original investigations of phytochemistry, pharmacology, toxicology, quality control, or ethnopharmacology of *R. parvifolius* with sufficient species-specific data.

### 2.3 Exclusion criteria

Conference abstracts without full text; duplicate reports; agronomic or ecological studies; studies on other *Rubus* species without *R. parvifolius*-specific data; reviews (used only to identify primary sources).

Study-selection flow (Figure 1, PRISMA-style): of 472 records initially identified across the four databases, 214 duplicates were removed; 258 records were screened by title and abstract, of which 149 were excluded after this stage; 109 full-text articles were assessed for eligibility, of which 52 were excluded after full-text review; 57 studies were included in the final synthesis. Many included studies contribute to more than one evidence domain (for example, phytochemistry and pharmacology); therefore the sum of studies counted within individual evidence domains exceeds the total number of included studies.

Studies were grouped qualitatively by pharmacological activity category and study type (*in vitro*, *ex vivo*, *in vivo* rodent, or clinical observation). Mechanistic findings are discussed with explicit distinction between pathway-associated expression changes and experimentally validated mechanisms. No formal risk-of-bias assessment or meta-analysis was performed.

We additionally evaluated each included primary study with attention to: (i) the adequacy of botanical identification (taxonomic authority, plant part, and geographic source); (ii) the level of chemical characterisation of the preparation tested (extract type, solvent, fractionation, and characterisation methods reported); (iii) the appropriateness of the pharmacological model and dose range—with *in vivo* extract doses reported in mg/kg/day and pure-metabolite concentrations in μmol/L, and with explicit flagging where extract doses exceed 1 g/kg/day; and (iv) the distinction between cell-free antioxidant assays (DPPH, ABTS, FRAP), which characterise extract chemistry but not pharmacological activity, and biologically relevant *in vitro* and *in vivo* bioassays. The findings of this assessment are discussed throughout Sections 6, 7.

## 3 Botanical characterisation and geographic distribution

### 3.1 Taxonomy and morphology

The species *R. parvifolius* L (synonym: *Rubus triphyllus* Thunb.) belongs to the tribe *Rubeae* within subfamily *Rosoideae* (Rosaceae). The species is placed within the subgenus *Idaeobatus*, sharing biosynthetic pathways with the pharmacologically well-studied congeners *Rubus idaeus* (raspberry) and *Rubus coreanus*. Morphologically, *R. parvifolius* is a weakly erect to arching deciduous shrub reaching 0.5–2.0 m, with recurved prickles and glandular hairs on stems and petioles. Leaves are ternate to pinnate with three to five leaflets, broadly ovate with serrate margins and stellate-pubescent abaxial surfaces. Flowers are actinomorphic, five-petalled, pink to pale purple (April–May). The aggregate raspberry-type fruit is 1–1.5 cm in diameter, ripening red-orange (June–July).

### 3.2 Geographic distribution and habitat

*Rubus parvifolius* is broadly distributed across eastern, central, and southern China—including Guangdong, Guangxi, Fujian, Zhejiang, Hunan, Jiangxi, Sichuan, and Yunnan—and extends to Japan, Korea, Taiwan, Vietnam, and northern India. Typical habitats include open hillsides, forest margins, field edges, and disturbed roadsides at low to mid elevations.

### 3.3 Botanical identification: macroscopic, microscopic, and molecular

Authentic identification of *R. parvifolius* is operationally important because the genus *Rubus* comprises approximately 700 species worldwide and approximately 200 in China, with morphological variation that can confound macroscopic identification—within the *Idaeobatus* subgenus and the closely related “hollow-stemmed *Rubus*” group with which several other Chinese medicinal *Rubus* species share habitat ranges. Lu et al. (Lu et al., 2024) published in 2024 a comprehensive identification protocol integrating four complementary methods, providing a framework relevant to quality-control standardisation:

*Macroscopic features*: distinctive recurved prickle morphology, leaf venation, and inflorescence architecture.

Microscopic features: Lu et al. reported that powdered root and stem of *R. parvifolius* contain calcium oxalate cluster crystals, calcium oxalate prismatic crystals, and bordered-pit vessels. Leaf tissue is characterised by two types of non-glandular trichome and one type of club-shaped glandular trichome (Lu et al., 2024).

Thin-layer chromatography (TLC): an effective method for routine quality identification of *R. parvifolius* root (Lu et al., 2024).

Molecular identification: internal transcribed spacer (ITS) sequence analysis can resolve 53 species within the hollow-stemmed *Rubus* group, providing reliable molecular discrimination from morphologically similar congeners. Lu et al. reported phylogenetic findings that diverge from traditional taxonomy, with the *velutiniformes* and *pubescentes* subgroups appearing as potential ancestors of other subgroups within this clade (Lu et al., 2024).

This four-tier identification framework supports the methodological foundation for *R. parvifolius* quality control and is referenced again in Section 8 in the context of pharmacopoeial standardisation gaps.

### 3.4 Chemotaxonomic position within *rubus*

Within the genus *Rubus*, *R. parvifolius* root is chemotaxonomically distinguished by a high relative abundance of ursane-type triterpenoid saponins—a feature shared with other *Rubeae* taxa but prominent in this species relative to fruit-derived *Rubus* preparations (Zhang et al., 2016b; Mei et al., 2016). Fruit-derived *Rubus* extracts (*R. idaeus* and *Rubus occidentalis*) are anthocyanin-dominated and are the chemical basis of cancer chemoprevention investigation in those species; the high root-saponin content of *R. parvifolius* shifts its pharmacological profile toward triterpenoid-mediated activities and may partly account for differences between the preclinical activities reported for *R. parvifolius* root preparations and those reported for fruit-derived congener extracts. This chemotaxonomic distinction is relevant when interpreting cross-species inference from *Rubus* genus-level reviews, which often conflate fruit and root data despite their different chemical profiles.

## 4 Ethnomedicinal background and traditional uses

### 4.1 Ethnomedical framework

Regional Chinese ethnomedical sources characterise *R. parvifolius* as sweet, bitter, and slightly cold (gān kǔwēi hán), with channel tropism to the bladder, lung, and liver meridians (terminology drawn from the broader East Asian herbal-medical framework). Principal traditional therapeutic actions include: clearing heat and resolving toxicity (qīng rè jiě dú); invigorating blood and resolving stasis (huó xuè huà yū); stopping bleeding (zhǐ xuè); relieving pain and swelling (xiāo zhǒng zhǐ tòng); and promoting bone regeneration (jiē gǔ shēng jī). Both oral preparations (6–15 g dried root, decocted in water) and external preparations (poultice, decoction wash) are documented (Chen and Mei, 2015; Guangdong Food and Drug Administration (compiled), 2019).

Core historical and authoritative sources include the Bencao Shiyi (Tang dynasty), the Quanguo Zhongcaoyao Huibian (1975), the Zhongyao Da Cidian (1985), and the Guangdong Provincial Standard for Chinese Medicinal Materials (Third Volume, 2019). The Mei Quanxi pharmacological compendium of Guangdong medicinal botanical drugs provides a focused regional review. A dedicated herbal-textual study by Chen XL and Mei QX (2015) (Chen and Mei, 2015) traces the historical use of *R. parvifolius* across classical materia medica, providing a consolidated source for the species’ traditional therapeutic indications and regional nomenclature.

### 4.2 Critical note: preparation–activity mismatch

A consideration of central importance—and one that is under-discussed in earlier reviews of this species—is that traditional preparations use aqueous decoction of dried root, whereas most modern experimental studies use 70% ethanol, n-butanol, or methanol extracts, or further purified fractions such as total saponins of *R. parvifolius* (TSRP). These preparation profiles are chemically distinct: aqueous decoction is expected to extract primarily saponin glycosides, flavonoid glycosides, polysaccharides, and organic acids, with limited extraction of lipophilic triterpenoid aglycones; alcohol-based and resin-purified preparations selectively enrich lipophilic and triterpenoid metabolites at the expense of polysaccharides and hydrophilic glycosides. The translational relevance of preclinical findings obtained with experimental extracts to traditional oral aqueous decoction therefore requires explicit caution. This consideration is returned to in Section 8.3.

### 4.3 Traditional indications and modern preclinical correspondence

The traditional therapeutic indications of *R. parvifolius* (root) recorded in the regional materia medica encompass five major functional categories: (i) activating blood circulation and resolving stasis (huó xuè huà yū—for traumatic injury, postpartum stasis, metrorrhagia, haematemesis); (ii) clearing heat and resolving toxicity (qīng rè jiě dú—for febrile illness, sore throat, dysentery, carbuncles, mumps); (iii) expelling wind and resolving dampness (qū fēng lì shī—for rheumatic-joint pain, damp-heat jaundice, damp-heat diarrhoea); (iv) promoting urination and resolving stranguria (lì shuǐ tōng lín—for oedema and urinary lithiasis); and (v) cooling the blood (liáng xuè—for haemoptysis, haematemesis, metrorrhagia). Table 1 summarises these traditional indications and the corresponding modern preclinical pharmacological evidence. The correspondence between traditional indications and modern preclinical findings is broad across most indication categories; however, this correspondence should be interpreted with the qualifications discussed in Section 10.2 (publication bias, preparation mismatch, model translation limits).

**TABLE 1:** Traditional therapeutic indications of *Rubus parvifolius* L. and corresponding modern preclinical pharmacological evidence.

Traditional indication	Traditional action	Modern evidence (model)	Proposed mechanism	Key references; evidence level
Haemorrhage	Haemostasis + blood activation (zhǐ xuè huó xuè)	Aqueous extract: reduced bleeding/clotting time and euglobulin lysis time; inhibited platelet thrombosis	Dual haemostatic-fibrinolytic activity (mechanism not fully characterised)	(Zhu et al., 1990); *in vivo* rodent/rabbit
Traumatic injury	Resolve stasis, promote healing (xiāo zhǒng jiē gǔ)	n-Butanol fraction: reduced carrageenan- and xylene-induced oedema (comparable to indomethacin); leaf > root > stem activity	NF-κB/COX-2/iNOS-associated changes; proposed contribution of leaf volatile oil	(Zhang et al., 2011; Yang et al., 2016); *in vivo* rodent
Hepatitis/liver disease	Clear heat, protect liver (qīng rè bǎo gān)	n-Butanol extract: improvement of CCl4-induced acute hepatic injury in mice; ALT/AST reduction; SOD maintenance; MDA reduction; histopathological improvement. Aqueous extract (ERPL): improvement of CCl4-induced chronic liver fibrosis in rats with concurrent p53 ↑, SLC7A11 ↓, GPX4 ↓ (ferroptotic signature)	Phenolic and triterpenoid fractions; ferroptotic mechanism (Wang Guo 2025)	(Gao et al., 2011; Wang et al., 2025); *in vivo* rodent
Cancer (nasopharyngeal, leukaemia)	Resolve toxicity, dispel masses (jiě dú sàn jié)	TSRP: dose-dependent A375 melanoma xenograft inhibition; K562 CML inhibition *in vitro* and xenograft; 2025 evidence for reversal of imatinib resistance in K562R cells *via* PI3K/AKT downregulation; single small clinical observation (n = 40, open-label randomised controlled trial) for peripheral neuropathy	Caspase-3/9 ↑; Bcl-2 ↓/Bax ↑; AMPK activation; STAT3/eIF4E associated changes; PI3K/AKT downregulation in resistance reversal	(Cheng et al., 2025; Zheng et al., 2007; Ge et al., 2014; Xu et al., 2018a; Xu et al., 2011; Zhang et al., 2014; Cao et al., 2021; Zhao and Ma, 2019; Xu et al., 2012); *in vitro* + *in vivo* rodent +1 small clinical observation
Nephritis, infection	Clear heat, drain dampness (qīng rè lì shī)	Leaf volatile oil: broad-spectrum antibacterial activity *in vitro* against Gram-positive, Gram-negative, and multi-drug-resistant clinical isolates; anti-inflammatory in LPS-stimulated macrophages	4-Hydroxy-3-methoxystyrene (dominant volatile metabolite); NO/iNOS-associated changes	(Cai et al., 2012; Kim et al., 2025); *in vitro* only
Cerebral disease (stroke, neuropathy)	Nourish blood, benefit sinews (yǎng xuè shū jīn)	TSRP and aqueous extract: reduction of cerebral infarct volume and improvement of neurological deficit in MCAO rat models (multiple groups); Suavissimoside R1 protects MPP + -injured dopaminergic neurons *in vitro* and improves striatal dopamine in MPTP-treated mice *in vivo*	Bcl-2 ↑/Bax ↓; oxidative-stress amelioration; dopaminergic neuroprotection of saponin (not aglycone) form	(Wang et al., 2006b; Wang et al., 2010; Wang et al., 2012; Chen et al., 2011); *in vivo* rodent (MCAO, MPTP)
Fatigue (tonic)	Supplement deficiency, boost vigour (bǔ yì fú zhèng)	Bioactivity-guided fractionation identified nigaichigoside F1 as the candidate active metabolite; TSRP extended swimming time to exhaustion, reduced serum lactate/urea nitrogen, preserved hepatic glycogen, and suppressed IL-6 and TNF-α	Anti-inflammatory and metabolic adaptation; nigaichigoside F1 identified as a candidate active metabolite	(Chen et al., 2013); *in vivo* rodent; bioactivity-guided fractionation

‘Evidence level’ refers to the highest level of evidence identified for each indication. All pharmacological evidence is preclinical or from very limited clinical observation; no randomised controlled trial has been published. Traditional preparations typically used aqueous decoction, which differs chemically from most experimental extracts (see Section 8.3).*.

## 5 Phytochemical composition

### 5.1 Overview

**FIGURE 2:**
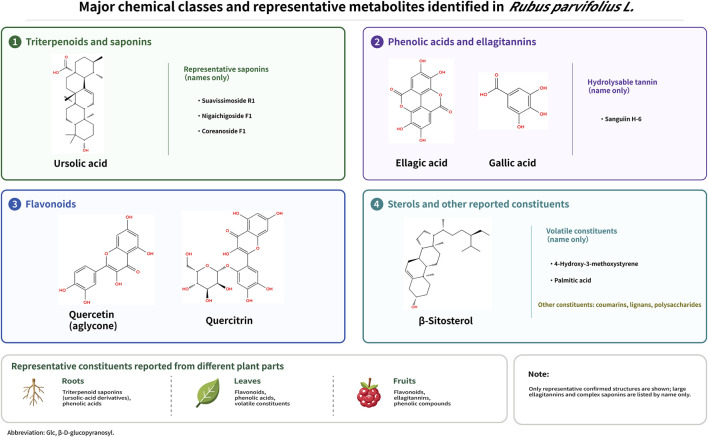
Major chemical classes and representative metabolites identified in *Rubus parvifolius L*. Four panels show: (1) triterpenoids and saponins—ursolic acid, with representative saponins listed by name (suavissimoside R1, nigaichigoside F1, coreanoside F1); (2) phenolic acids and ellagitannins—ellagic acid and gallic acid, with the hydrolysable tannin sanguiin H-6 listed by name; (3) flavonoids—quercetin (aglycone) and quercitrin (glycoside); and (4) sterols and volatile constituents—β-sitosterol, with volatile constituents listed by name (4-hydroxy-3-methoxystyrene, palmitic acid) and other reported constituents (coumarins, lignans, polysaccharides). The bottom panel summarises representative constituents reported from roots, leaves, and fruits. Only representative confirmed structures are shown; large ellagitannins and complex saponins are listed by name only. Abbreviation: Glc, β-D-glucopyranosyl.

Systematic phytochemical investigation of R. parvifolius (1988–2026) has identified more than 60 individual metabolites across several major structural classes, dominated by ursane- and oleanane-type pentacyclic triterpenoids and their corresponding saponins. Other reported classes include flavonoids and flavonol glycosides, phenolic acids and ellagitannins, volatile constituents, polysaccharides, sterols, and minor classes such as sphingolipids, anthraquinones, and trace elements. The major metabolite classes are summarised in Figure 2.

### 5.2 Triterpenoids and triterpenoid saponins (dominant class)

The first triterpenoid isolation from this species was reported by Choi et al., in 1991 (Choi et al., 1991), who obtained euscaphic acid, 2α-hydroxyursolic acid, and tormentic acid from the root. Wang et al. (1994) subsequently reported the first isolation of niga-ichigoside F1 and suavissimoside R1 from *R. parvifolius* roots—two triterpenoid saponins later confirmed as candidate active metabolites (Section 6). Zhang et al. (2016a) isolated 19 metabolites including 12 triterpenoids from the ethanolic root extract, with several first reports for *R. parvifolius*, the genus *Rubus*, and Rosaceae (full inventory in Table 2); two new oleanane-type triterpenoids—parvifolactone A and rubuside P—were subsequently reported by Zhang et al. (2016) alongside 11 known triterpenoids (Zhang et al., 2016b).

**TABLE 2:** Major chemical metabolites of *Rubus parvifolius* L (selected representative metabolites).

Metabolite	Structural class	Plant part	Isolation method	Analytical method	Pharmacological notes	Key references
Ursolic acid	Ursane triterpenoid	Root	Column chromatography	NMR; HPLC	Reported metabolite; pharmacological relevance requires direct validation	Du and Rao (2003), Mei et al. (2016)
2α-Hydroxyursolic acid	Ursane triterpenoid	Root	Column chromatography	NMR	*In vitro* HepG2 cytotoxicity (∼43% inhibition at 10 μmol/L × 72 h)	Choi et al. (1991), Zhang et al. (2016a)
2-Oxopomolic acid	Ursane triterpenoid	Root	Column chromatography	NMR	RAW264.7 NO inhibition ∼45% at 10 μmol/L	Zhang et al. (2016a), Zhang et al. (2016b)
Tormentic acid	Ursane triterpenoid	Root	Column chromatography	NMR	RAW264.7 NO inhibition ∼31% at 10 μmol/L	Choi et al. (1991), Zhang et al. (2016a)
Euscaphic acid	Ursane triterpenoid	Root	Column chromatography	HPLC	Reported metabolite	Du and Rao (2003)
Parvifolactone A*	Triterpenoid lactone (novel)	Root	Column chromatography	NMR; HR-MS	First-reported; novel oleanane lactone	Zhang et al. (2016b)
Rubuside P*	Oleanane triterpenoid saponin (novel)	Root	Column chromatography	NMR; HR-MS	First-reported	Zhang et al. (2016b)
Maslinic acid	Oleanane triterpenoid	Root	Column chromatography	NMR	First-reported in this species	Zhang et al. (2016b)
Suavissimoside R1	Ursane triterpenoid saponin	Root	Column chromatography; prep. HPLC	NMR	Dopaminergic neuroprotection *in vitro* and *in vivo*; activity requires sugar moiety	Zhang et al. (2016b), Chen et al. (2011)
Nigaichigoside F1	Ursane triterpenoid saponin	Root	Column chromatography	NMR	Candidate anti-fatigue metabolite (bioactivity-guided fractionation)	Zhang et al. (2016b), Chen et al. (2013)
Coreanoside F1	Triterpenoid saponin dimer	Root	Column chromatography	MS	Anti-fatigue (*in vivo*); co-active with above	Zhang et al. (2016b)
Ellagic acid	Polyphenol/ellagitannin hydrolysate	Root; Fruit; Leaf	n-Butanol extraction	HPLC-MS/MS	Antioxidant; anti-inflammatory (*in vitro*)	(Du et al., 2005; Gao et al., 2011)
Sanguiin H-6	Dimeric ellagitannin	Root	Column chromatography	HPLC-MS	Anti-osteoclastogenic (RANKL pathway); breast cancer-relevant activity in cross-species studies	Sakai et al. (2016), Park et al. (2017), Park et al. (2016)
Gallic acid	Hydroxybenzoic acid	Root	Ethanol extraction	HPLC-MS	Antioxidant; AChE inhibition (*in vitro*)	Lin et al. (2021)
(+)-Catechin/L-Epicatechin	Flavan-3-ol	Root; Leaf	Column chromatography	NMR; HPLC	Antioxidant; first reported by Do et al., 1988	Huang et al. (2016), Do et al. (1988)
Quercetin 3,7-diglucoside	Flavonol diglycoside	Leaf	LC-MS/MS phytochemical profiling	LC-MS/MS; HPLC	Dominant leaf flavonoid (12.73 mg/g DW); anti-inflammatory (cell-based)	Kim et al. (2025)
Hirsutrin	Flavonol glycoside	Leaf	LC-MS/MS phytochemical profiling	LC-MS/MS	Leaf-localised (4.74 mg/g DW); antioxidant; anti-inflammatory (cell-based)	Kim et al. (2025)
Quercitrin	Flavonol glycoside	Root; Leaf	Column chromatography	HPLC-ESI-MS	Antioxidant (*in vitro*)	Wang et al. (2006a)
Procyanidin B3/C2	Proanthocyanidin oligomer	Root	UPLC-MS/MS profiling	UPLC-MS/MS	Identified in 2025 UPLC-MS/MS profile of root	Jia et al. (2025)
(E)-3-Indoleacrylic acid	Indole derivative	Root	UPLC-MS/MS profiling	UPLC-MS/MS	Identified in 2025 UPLC-MS/MS profile; network pharmacology target	Jia et al. (2025)
Caprolactam	Lactam	Root	UPLC-MS/MS profiling	UPLC-MS/MS	Identified in 2025 UPLC-MS/MS profile; network pharmacology target	Jia et al. (2025)
4-Hydroxy-3-methoxystyrene	Phenolic ether	Leaf VO	Steam distillation	GC-MS	Dominant constituent detected in leaf volatile oil; antibacterial activity reported *in vitro*	Cai et al. (2012)
Palmitic acid	Fatty acid	Leaf VO	Steam distillation	GC-MS	Fatty acid detected in leaf VO GC-MS profile	Tan et al. (2003a)
β-Sitosterol; β-Daucosterol; Stigmasterol; Campesterol	Phytosterol	Root	Column chromatography	NMR; TLC	Co-occurring root sterols	Liang et al. (2011), Do et al. (1988)

New metabolites first reported from this species.

Bioactivity-guided fractionation of TSRP identified nigaichigoside F1, suavissimoside R1, and coreanoside F1 as candidate active saponins (Chen et al., 2013), with nigaichigoside F1 specifically identified as the principal anti-fatigue metabolite. Suavissimoside R1 has been independently characterised as a dopaminergic neuroprotective metabolite, with the sugar moiety shown to be essential for activity (Chen et al., 2011).

### 5.3 Phenolic acids and ellagitannins

Phenolic metabolites are well-represented across plant parts. Gao et al. (2011) characterised *R. parvifolius* root n-butanol extract by HPLC-MS/MS, identifying caffeic acid conjugates and ellagic acid. Lin et al. (2021) characterised gallic acid and other phenolic metabolites of root extracts with *in vitro* α-glucosidase and acetylcholinesterase inhibitory activities. The dimeric ellagitannin sanguiin H-6 is a particularly notable bioactive metabolite: Sakai et al. (2016) characterised sanguiin H-6 from *R. parvifolius* as an inhibitor of RANKL-induced osteoclastogenesis. Cross-species data for sanguiin H-6 from other *Rubus* species (Park et al., 2017; Park et al., 2016) are discussed with appropriate caution in Section 6.1.3.

A complementary thesis investigation by Hu (2013) characterised several additional metabolites from stems and leaves, including diterpenoid glycosides and triterpenoid glycosides, further expanding the known chemical diversity of *R. parvifolius*.

### 5.4 Flavonoids and flavonol glycosides

Flavonoid composition of *R. parvifolius* has been characterised across plant parts, with quercitrin and other flavonol glycosides reported from both root and leaf (Wang Y. et al., 2006). Kim et al. (2025) quantified leaf flavonoids in their 2025 atopic dermatitis study, identifying quercetin 3,7-diglucoside as the dominant leaf flavonoid (12.73 mg/g DW), followed by hirsutrin (4.74 mg/g DW); ellagic acid (a phenolic constituent; Section 5.3) was co-quantified in the same leaf fraction. Catechins—including (−)-epicatechin from fresh roots (Do et al., 1988) and (+)-catechin reported as a first-species record (Liang et al., 2011) — have also been quantified across root, stem, and leaf tissues by validated HPLC methods (Huang et al., 2016), informing the part-specific bioactivity differences discussed in Section 6.5.

### 5.5 Volatile oils (leaf-dominant)

Two GC-MS investigations have characterised the leaf volatile oil. Tan et al. (2003a) identified 20 components in the steam-distilled fraction, including palmitic acid as a fatty acid detected in the GC-MS profile. Cai et al. (2012) applied optimised hydro-distillation conditions, identifying 30 metabolites with 4-hydroxy-3-methoxystyrene as the dominant metabolite (66% of total volatile oil) and broad-spectrum antibacterial activity *in vitro* (Section 6.6).

### 5.6 Polysaccharides, sterols, sphingolipids, and other metabolites

Crude root polysaccharides have been characterised by aqueous extraction and ethanol precipitation (Hu, 2011). Trace element analysis identified iron, manganese, zinc, and copper at regional pharmacological interest (Wei et al., 1996). β-Sitosterol and β-daucosterol are the major root sterols (Liang et al., 2011), with additional phytosterols (stigmasterol, campesterol) reported by Do et al. (1988). A phytochemical investigation by Mei et al. (2016) further isolated twelve metabolites including one ceramide (first sphingolipid from Rosaceae), two anthraquinones, four triterpenoids, two flavonoids, one furan derivative, one fatty acid ester, and two sterols; metabolites two to five are first records from the genus *Rubus*. The pharmacological significance of these less-investigated metabolite classes in *R. parvifolius* remains unclear.

### 5.7 Modern integrated characterisation: UPLC-MS/MS and network pharmacology

Jia et al. (2025) reported an integrated UPLC-MS/MS plus network pharmacology characterisation of *R. parvifolius* root directed at the traditional indications of fever, sore throat, and inflammation, identifying 20 root metabolites (Table 2) and 24 core targets with AKT1 and TNF as principal hubs. These network pharmacology findings are hypothesis-generating and require experimental validation.

### 5.8 Chemotaxonomic synthesis

The *R. parvifolius* phytochemical profile is dominated by ursane- and oleanane-type pentacyclic triterpenoids—consistent with the *Rubeae* chemotaxonomic position but with higher root saponin abundance than the fruit-dominated *Rubus* congeners used in chemoprevention research. Differences between traditional aqueous decoctions and experimentally enriched saponin fractions should be considered when interpreting pharmacological findings (Sections 4.2, 8.3, 10).

Several metabolites reported from *R. parvifolius*—including β-sitosterol, ursolic acid, quercetin, and gallic acid—are ubiquitously distributed across plant families; their presence in an extract cannot be assumed to account for pharmacological activity without direct experimental validation. We therefore distinguish between (a) marker metabolites for chemical characterisation and quality control, and (b) candidate active metabolites supported by species-specific bioactivity-guided evidence (principally suavissimoside R1, nigaichigoside F1, sanguiin H-6, and selected ursane triterpenoids; see Sections 5.2, 6).

## 6 Pharmacological activities

**FIGURE 3:**
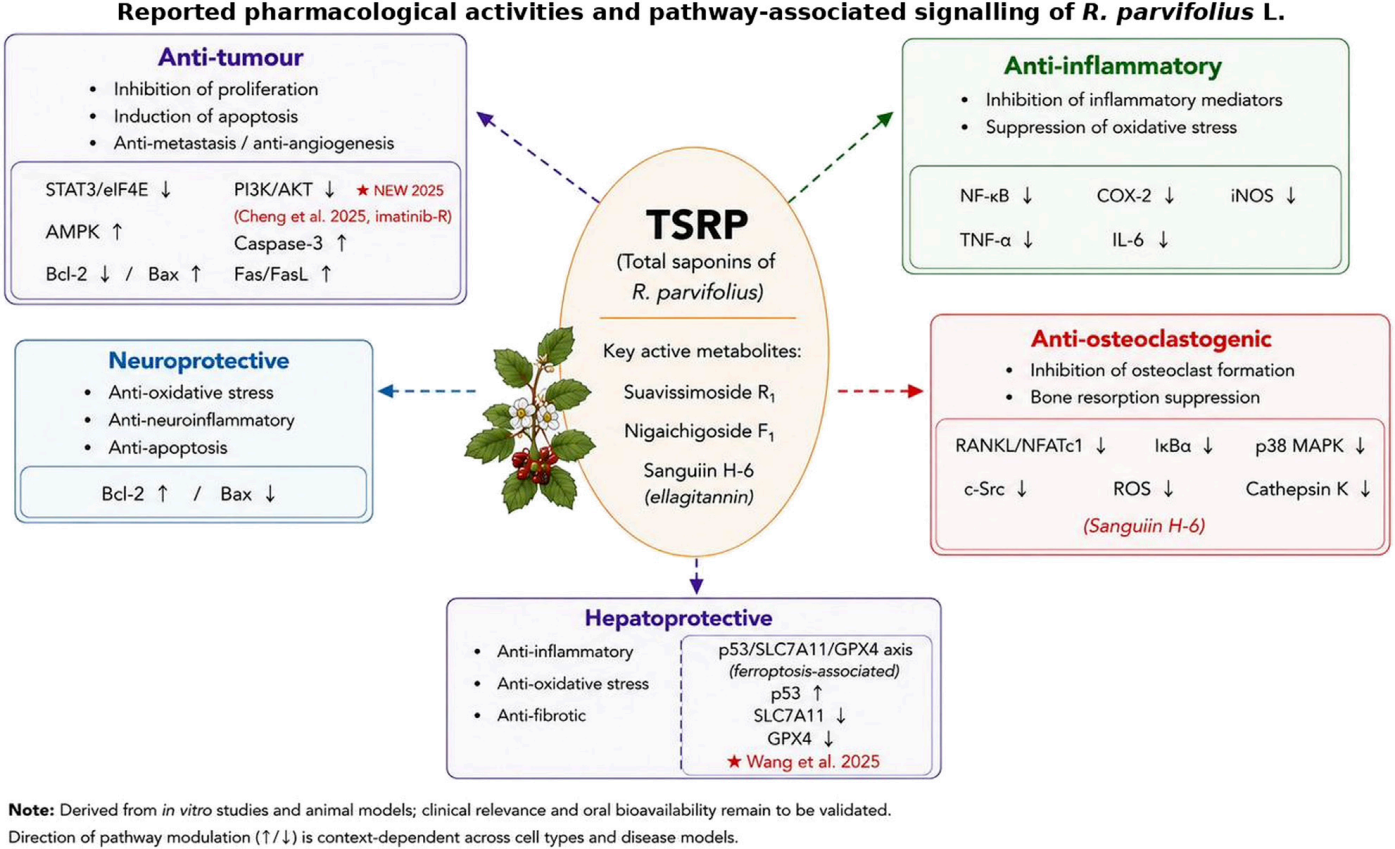
Reported pharmacological activities and pathway-associated signalling of *Rubus parvifolius* L. Five activity panels are shown: anti-tumour (upper-left, including STAT3/eIF4E and PI3K/AKT downregulation, AMPK activation, Bcl-2/Bax shift, Fas/FasL induction; ★ new 2025 evidence for imatinib-resistance reversal), anti-inflammatory (upper-right, NF-κB, COX-2, iNOS, TNF-α, IL-6 downregulation), neuroprotective (left, anti-apoptotic Bcl-2/Bax shift), anti-osteoclastogenic (right, mediated by sanguiin H-6), and hepatoprotective (bottom, including p53/SLC7A11/GPX4 ferroptotic axis; ★ new 2025 evidence). TSRP, total saponins of *Rubus parvifolius*. Solid arrows denote reported pharmacological activities; dashed arrows denote pathway-associated signalling. Directional annotations (↑/↓) reflect reported expression or activity changes rather than experimentally validated mechanisms.

A schematic overview of the principal pharmacological activities of *R. parvifolius*, candidate active metabolites, and reported pathway associations is provided in Figure 3.

The following sections summarise the pharmacological evidence across the eight activity categories with substantive preclinical literature, noting study type and key strengths and limitations for each. Unless otherwise stated, the reported pathway changes are associations rather than mechanisms validated through knockdown, rescue, or genetic models (see Section 7.3).

### 6.1 Anti-tumour activity

#### 6.1.1 Melanoma

Cao et al. (2021) and Zhao and Ma (2019) characterised TSRP-mediated inhibition of A375 malignant melanoma cell proliferation, invasion, and migration *in vitro*, and confirmed dose-dependent inhibition of xenograft growth (TSRP 25–100 mg/kg) in an A375 nude-mouse xenograft model, together with significant reduction of lung metastatic foci in an A375 tail-vein metastasis model. Methodological limitations include modest group sizes, intraperitoneal administration (limiting translation to clinical oral use), absence of pharmacokinetic data, and absence of orthotopic model validation.

Zheng et al. (2007) further characterised TSRP activity against Hut-78 (cutaneous T-cell lymphoma) and HR8348 (rectal adenocarcinoma; G2/M arrest via CDK1/cyclin B1 expression changes), but reported no significant activity against A431 — indicating tumour-type selectivity, the mechanistic basis of which has not been established.

#### 6.1.2 Leukaemia and imatinib resistance reversal

The leukaemia literature for this species includes multiple independent *in vitro* and *in vivo* studies. Initial characterisation of *R. parvifolius* and its total saponins on K562 cells *in vitro* and *in vivo* was reported by Xu et al. (2011) in 2011. Ge et al. (2014) subsequently characterised TSRP activity against K562 chronic myeloid leukaemia (CML) cells, with associated changes in AMP-activated protein kinase (AMPK) activation, signal transducer and activator of transcription 3 (STAT3) Tyr705 phosphorylation reduction, Caspase-3/9 increases, and Bcl-2 reduction. The K562 mechanistic findings were subsequently extended *in vivo* by Xu et al. (2018a) (K562 xenograft model in nude mice; control, cytosine arabinoside, and three TSRP groups [20, 40, 100 mg/kg], with anti-tumour rates and STAT3/eIF4E immunohistochemistry changes characterised) and by Zhang et al. (2014) in a complementary *in vivo* CML xenograft study. The leukaemia activity profile has been extended beyond K562 cells: Xu et al. (2018b) characterised dose-dependent TSRP-induced apoptosis in HL-60 cells at 200, 400, and 800 mg/L, with concurrent Bcl-2, Bax, Caspase-3, Caspase-9, and Fas mRNA expression changes proposing dual Bcl-2 and Fas pathway involvement. TSRP-induced apoptosis was also reported in lymphoma cell lines through upregulated Bax/Fas and downregulated Bcl-2 expression (Xu et al., 2019). A separate study by the same group (Xu et al., 2015) characterised synergistic apoptosis induction by TSRP in combination with homoharringtonine or cytarabine in K562 cells, with Western blot evidence for enhanced cleaved Caspase-3/9, cleaved PARP, and reduced Survivin expression compared with single-agent treatment—providing preclinical rationale for combination chemotherapy strategies.

In a 2025 study, Cheng et al. (2025) characterised TSRP in an imatinib-resistant K562R cell line. The combination of TSRP and imatinib produced significantly greater inhibition of cell viability and apoptosis than either single agent (P < 0.01 versus control), with significant downregulation of phosphorylated PI3K and AKT in the combination group; the authors propose that TSRP reverses imatinib resistance through the PI3K/AKT pathway, a finding of potential relevance in imatinib-resistant CML models that requires further validation.

A small clinical study by Xu et al. (2012) (n = 40, open-label randomised controlled, two-arm) reported reduced bortezomib-induced peripheral neuropathy in multiple myeloma patients receiving *R. parvifolius* decoction combined with vitamin B12. This study provides only a preliminary clinical signal; it cannot establish efficacy or safety given its design limitations, and is included here principally to delineate the present limits of the clinical evidence base.

#### 6.1.3 Breast cancer: a research gap

Direct anti-breast-cancer evidence specifically for *R. parvifolius* preparations has not yet been published. Cross-species data for sanguiin H-6 — an ellagitannin present in *R. parvifolius* root (Sakai et al., 2016) and in other *Rubus* species—provide indirect mechanistic rationale: Park et al. (2017) reported cytotoxicity against MCF-7 and MDA-MB-231 breast cancer cells through an oestrogen receptor-independent mechanism, and Park et al. (2016) (different research group) reported anti-metastatic activity in MDA-MB-231 cells via VEGF/Akt/ERK1/2 signalling, although in their study sanguiin H-6 was isolated from *Rubus coreanus*. The relative abundance of sanguiin H-6 in *R. parvifolius* versus other *Rubus* preparations has not been quantitatively characterised, and whole-root or TSRP extracts have not been tested in breast cancer models. Direct testing of chemically characterised *R. parvifolius* preparations is a research priority; the cross-species data should not be read as establishing activity for this species.

### 6.2 Neuroprotective activity

The neuroprotective evidence base for *R. parvifolius* spans both stroke and Parkinson’s disease models with consistent positive preclinical findings across multiple research groups.

In stroke models, Wang JS. et al. (2006), Wang et al. (2010), Wang et al. (2012) systematically characterised TSRP neuroprotection in middle cerebral artery occlusion (MCAO) rat models: reduced infarct volume, improved neurological deficit scores, reduced neuronal apoptosis, and Bcl-2/Bax protein and mRNA expression changes, with oxidative stress marker amelioration. Complementary mechanistic work by the same group characterised TSRP-mediated reduction of cerebral oedema and modulation of blood–cerebrospinal fluid barrier permeability in MCAO rats (Wang et al., 2007), providing further evidence for the cerebrovascular protective profile. These findings have been independently replicated across multiple research groups. Methodologically, these are reasonably well-characterised rodent studies; the principal translational caveat is the limited predictive validity of MCAO models for clinical stroke outcomes.

In Parkinson’s disease (PD) models, the dopaminergic-protective metabolite of *R. parvifolius* root was first identified by Yu et al. (2008) in 2008 through bioactivity-guided fractionation against MPP + -injured dopaminergic neurons, isolating suavissimoside R1 as the candidate active metabolite. This early identification was subsequently extended mechanistically by Chen et al. (2011) who characterised dopaminergic neuroprotection by suavissimoside R1 and demonstrated that this activity depends on the intact glycoside form: alkaline hydrolysis to the aglycone abolished activity. The intact glycoside protected MPP + -injured rat primary mesencephalic dopaminergic neurons at 100 μmol/L and 300 μmol/L (*in vitro*) and increased striatal dopamine content in MPTP-treated mice at 0.75 mg/kg (*in vivo*); the aglycone showed no protective activity at any tested dose. This study directly compared the intact glycoside with its aglycone and represents an early characterisation of dopaminergic neuroprotective activity for a *Rubus* ursane saponin in both *in vitro* and *in vivo* PD models.

Together, the stroke and PD evidence supports modest preclinical credibility for *R. parvifolius* neuroprotection. Translation remains highly uncertain in the absence of pharmacokinetic data and in light of the considerable preclinical-to-clinical attrition rates typical of natural-product neuroprotection.

### 6.3 Hepatoprotective and anti-hepatic-fibrosis activity

Two distinct lines of hepatoprotective evidence have emerged for *R. parvifolius*, differing in extract type, model, and proposed mechanism.

#### 6.3.1 Acute hepatic injury (CCl4)

Gao et al. (2011) characterised hepatoprotection of the n-butanol extract of *R. parvifolius* root in male ICR mice with CCl4-induced acute liver injury. The extract significantly attenuated CCl4-induced elevations of serum ALT and AST, prevented the CCl4-induced reduction of hepatic SOD activity, reduced hepatic MDA content, and improved histopathology compared with the CCl4 control. Phytochemical analysis by HPLC-MS/MS and offline FT-ICRMS identified phenolic metabolites including caffeic acid conjugates and ellagic acid as the major compositional contributors, leading the authors to propose phenolic antioxidant activity as a principal mechanism. The study uses a single dose-comparison design; dose-response characterisation across the same model would aid translational interpretation.

A separate thesis study by Zhou (2010) reported preliminary *in vivo* anti-hepatitis B virus activity of *R. parvifolius* extracts; this remains unpublished in peer-reviewed form and is included here only to indicate the scope of preliminary investigation.

#### 6.3.2 Chronic liver fibrosis and ferroptosis (2025)

A 2025 study by Wang et al. (2025) characterised the aqueous extract of *R. parvifolius* root (ERPL) in a CCl4-induced chronic liver fibrosis model in SD rats (oral gavage, two doses). Compared with the model group, both ERPL doses significantly reduced hepatic inflammatory infiltration and fibrosis (HE and Masson staining) and lowered serum ALT, AST, ALP, laminin, PIIINP, and type IV collagen (all P < 0.05). Western blot showed increased p53 and decreased SLC7A11 and GPX4 expression—a signature consistent with ferroptosis induction—leading the authors to propose that ERPL ameliorates hepatic fibrosis via p53/SLC7A11/GPX4-mediated ferroptotic signalling.

This extends *R. parvifolius* pharmacology into ferroptosis-associated signalling; as elsewhere, these are pathway associations, and ferroptosis-inhibitor co-treatment or SLC7A11/GPX4 knockdown-rescue would be needed to establish mechanism.

### 6.4 Antioxidant activity

Flavonoid- and phenolic-rich fractions of *R. parvifolius* show radical-scavenging activity in chemical assays. Zheng et al. (2013) characterised dose-dependent *in vitro* radical-scavenging activity (DPPH, ABTS, hydroxyl radical assays) of flavonoid fractions from root, stem, and leaf, with leaf fraction showing the greatest activity. We note that DPPH, ABTS, FRAP and related radical-scavenging assays are chemical antioxidant capacity measurements and are not by themselves accepted as pharmacological evidence; these results are therefore reported here as chemical profile characterisations rather than as pharmacological mechanisms (see also Section 7). Lin et al. (2021) additionally characterised α-glucosidase and acetylcholinesterase inhibitory activities of root extract *in vitro*, with potential relevance to diabetes and neurodegeneration research lines.

*In vivo* antioxidant activity in lead-intoxicated rats was reported by Liang et al. (2006) with elevated serum SOD activity and reduced hepatic MDA. This represents one of the few *in vivo* antioxidant characterisations for *R. parvifolius* in a disease-relevant model, although the chemical fraction tested was not fully characterised.

### 6.5 Anti-inflammatory activity

Anti-inflammatory activity has been characterised at multiple levels. Zhang et al. (2011) demonstrated dose-dependent reduction of carrageenan-induced paw oedema and xylene-induced ear oedema in rodents by the n-butanol fraction of *R. parvifolius*, with effect magnitudes comparable to indomethacin in the conditions tested. Yang et al. (2016) published a comparison of root, stem, and leaf aqueous extracts of *R. parvifolius* (three doses each: 18, 12, 6 g·kg^-1^) in carrageenan-induced paw oedema and cotton-pellet-induced granuloma models in SD rats. The activity ordering across both models was consistent: leaf > root > stem. The high- and mid-dose leaf extract groups produced significant reductions of paw oedema at 2 and 3 h; the high-dose root extract group produced significant reductions at 2 h; the cotton-pellet granuloma weight was significantly reduced by all groups except the low-dose root group, with the same activity ordering. The authors propose that leaf volatile oil—known to be dominated by 4-hydroxy-3-methoxystyrene (Cai et al., 2012) — may contribute to leaf anti-inflammatory activity. This study provides experimental basis for plant-part selection in clinical formulation and broadens the typically root-focused pharmacological evaluation.

Cell-based anti-inflammatory characterisation has been informative for specific triterpenoid metabolites. Zhang et al. (Zhang et al., 2016a) tested 12 triterpenoids from *R. parvifolius* root in lipopolysaccharide (LPS)-induced RAW264.7 macrophages at 10 μmol/L for 24 h. Two triterpenoids inhibited NO production: 2-oxopomolic acid (∼45% inhibition) and tormentic acid (∼31% inhibition). This provides specific structure-activity information that links the anti-inflammatory profile of TSRP to identifiable triterpenoid metabolites.

Kim et al. (2025) reported anti-inflammatory activity of *R. parvifolius* leaf extract in HaCaT keratinocytes and proposed atopic dermatitis as a candidate application, with characterisation of quercetin 3,7-diglucoside as the dominant flavonoid metabolite.

### 6.6 Antibacterial activity

Leaf volatile oil demonstrates broad-spectrum antibacterial activity *in vitro*. An earlier essential oil characterisation by Tan et al. (2003b) documented antibacterial activity of *R. parvifolius* leaf and root essential oils against *Staphylococcus aureus* and *Escherichia coli*. Cai et al. (2012) subsequently reported MIC values against representative Gram-positive and Gram-negative pathogens and multi-drug-resistant clinical isolates; the leaf volatile oil was inhibitory at concentrations of clinical interest for topical applications. The dominant volatile metabolite — 4-hydroxy-3-methoxystyrene (66% of total volatile oil) — is proposed as the principal antibacterial metabolite. Root volatile oil showed no significant antibacterial activity in the same study, again consistent with the part-specific bioactivity pattern documented by Yang et al. (2016).

Notes on translational interpretation: (i) the antibacterial concentrations characterised are plausibly achievable for topical application but are not achievable systemically by oral administration without pharmacokinetic data; (ii) the volatile metabolite profile means that systemic exposure would be highly route-dependent; (iii) the antibacterial finding is most relevant to traditional external preparations (poultices, washes) and would require dedicated formulation development for topical clinical investigation.

### 6.7 Anti-fatigue activity

Chen et al. (2013) characterised anti-fatigue activity of R. parvifolius by weight-loaded forced swimming in mice, using bioactivity-guided fractionation (macroporous resin, 50% ethanol elution) to obtain three saponins—nigaichigoside F1, suavissimoside R1, and coreanoside F1 — and identifying nigaichigoside F1 as the candidate active metabolite. The TSRP fraction extended time to exhaustion, reduced serum urea nitrogen and lactate, increased hepatic glycogen, limited the rise in serum lactate dehydrogenase, and suppressed serum IL-6 and TNF-α, consistent with combined metabolic and anti-inflammatory effects.

### 6.8 Haemostatic-fibrinolytic and anti-osteoclastogenic activity

Zhu et al. (1990) documented dual haemostatic-fibrinolytic activity of *R. parvifolius* aqueous extract—shortening bleeding/clotting time in mice, shortening euglobulin lysis time in rabbits, inhibiting platelet thrombosis *in vivo*, increasing coronary flow in isolated rat heart, and increasing tolerance to hypoxia. This early report is consistent with the traditional principle of harmonising haemostasis and blood activation (zhǐ xuè huó xuè) recorded in regional Chinese ethnomedicine and remains a foundational reference for the cardiovascular and haemostatic profile of this species.

Sakai et al. (2016) characterised sanguiin H-6 from *R. parvifolius* as an inhibitor of RANKL-induced osteoclastogenesis, with associated changes in IκBα, p38 MAPK, NFATc1, cathepsin K, c-Src, and reactive oxygen species in both *in vitro* and *in vivo* TNF-α-induced osteoclast models. Independent characterisation of sanguiin H-6 anti-osteoclast activity in other contexts supports the biological plausibility of this finding. The work has relevance to cancer skeletal metastasis and to osteoporosis.

## 7 Strength and limitations of current evidence

This section provides a qualitative cross-cutting appraisal of evidence quality across the pharmacological literature reviewed in Section 6 (summarised in Table 3)—a critical assessment step absent from earlier *R. parvifolius* reviews.

**TABLE 3:** Evidence strength and limitations summary, by pharmacological activity Evidence strength assessment across all pharmacological activity categories with substantive *Rubus parvifolius*-specific preclinical literature, drawn from n = 57 included studies (Figure 1). ‘Translational risk’ reflects key gaps in pharmacokinetic data, formulation standardisation, and clinical evidence (see Section 7).

Activity	Highest evidence level available	No. Independent studies/Groups	Key methodological limitations	Translational risk	Assessment
Anti-melanoma	*In vivo* rodent xenograft + metastasis model	1 *in vivo*; multiple *in vitro*	Modest group sizes; intraperitoneal route; no pharmacokinetic data; no orthotopic model	High — route, dose uncertain	Requires dose-finding, pharmacokinetic assessment, and oral-route validation
Anti-leukaemia (incl. imatinib resistance)	*In vivo* rodent; limited clinical observation only for treatment-related peripheral neuropathy, not anti-leukaemia efficacy	3+ *in vivo* (Ge, Xu, Cheng); the single available clinical observation (Xu et al., 2012) (n = 40, open-label randomised controlled trial) addressed bortezomib-induced peripheral neuropathy in multiple myeloma	K562 cell line single-source; pathway associations not formally validated; no clinical anti-leukaemia data	Moderate — preclinical evidence requires direct clinical validation	Preclinical hypothesis-generating; no clinical anti-leukaemia efficacy demonstrated to date
Anti-breast-cancer (hypothesis-generating)	Other *Rubus* species *in vitro* data for sanguiin H-6	0 direct studies for *R. parvifolius*; 2 cross-species *in vitro*	No direct *R. parvifolius* test in breast cancer models; cross-species inference only	Very high — no direct evidence	Hypothesis-generating only
Neuroprotection (MCAO; PD)	*In vivo* rodent (MCAO and MPTP)	4+ groups (MCAO); 1 group (PD)	MCAO has low human translation rate; PD model single research group; no PK	Very high	Consistent rodent findings; moderate preclinical credibility; clinical translation highly uncertain
Hepatoprotection (acute CCl4)	*In vivo* rodent	1	Single-dose-class comparison; no formal dose-response	Moderate	Moderate evidence; would benefit from dose-response and chronic model — partially addressed by ferroptosis study
Anti-hepatic-fibrosis (chronic, 2025)	*In vivo* rodent	1	Single research group; ferroptotic mechanism is pathway association	Moderate-high	Requires independent replication and formal ferroptosis-pathway validation
Antioxidant	*In vitro* + *in vivo* rodent (Pb-intoxication)	Multiple *in vitro*; 1 *in vivo*	Cell-free assays predominate; no clinical-translation pathway	High	Well-established *in vitro*; no clinical relevance demonstrated
Anti-inflammatory (part-specific)	*In vivo* rodent + *in vitro* structure-activity	2 *in vivo* (whole extract); 1 structure-activity (triterpenoid)	No formal pathway validation; multiple cell lines	Moderate-high	Mechanistic associations only; supported by structure-activity data
Antibacterial	*In vitro* (leaf volatile oil)	1 comprehensive	No *in vivo* infection model; topical/external relevance only	Very high (systemic); Moderate (topical)	Relevant for topical/external preparations; no systemic evidence
Anti-fatigue	*In vivo* rodent + bioactivity-guided fractionation	1	Forced-swimming model translation uncertain; biomarker mechanism only	Moderate-high	Bioactivity-guided fractionation with active-metabolite identification; specific active metabolite identified
Anti-osteoclastogenic	*In vitro* + *in vivo* (TNF-α model)	1 (Sakai)	Single research group; specific to RANKL/TNF-α pathway	Moderate-high	*In vitro* and *in vivo* pathway-associated evidence; potential relevance to bone-resorption disorders

### 7.1 Cross-cutting limitations

Five cross-cutting limitations affect the entire R. parvifolius preclinical literature and warrant explicit acknowledgment:

- Pharmacokinetics—summary, with full discussion in Section 7.2 below. No pharmacokinetic study has been published for any *R. parvifolius* metabolite following oral administration in any species. This is a major translational gap, addressed as a dedicated subsection below.
- Toxicology. No formal subchronic (90-day), chronic, genotoxicity, reproductive, or developmental toxicity study has been published for any *R. parvifolius* extract or metabolite. Available acute toxicity data are limited to TSRP up to 3.0 g/kg (intraperitoneal) and 10 g/kg (intragastric) in mice without observable toxicity (Wang et al., 2010); therapeutic doses in efficacy studies (5–100 mg/kg) are far below these acute thresholds. The traditional safety record is generally favourable; pregnancy contraindication is noted in the Guangdong monograph based on traditional experience. Regulatory-grade toxicology to IND-enabling standards has not been conducted. The absence of published toxicological data should not be interpreted as evidence of safety.
- *Extract heterogeneity.* TSRP batch composition is not standardised across research groups; extraction solvents range from aqueous decoction (traditional) through 70% ethanol, n-butanol, methanol, and resin-purified saponin fractions. Inter-study comparability is uncertain, and pharmacological extrapolation across preparation types is not warranted without explicit phytochemical comparison. The 2025 integrated UPLC-MS/MS and network pharmacology study (Jia et al., 2025) provides a valuable framework for future extract characterisation.
- *Mechanistic association* versus *established mechanism.* The mechanistic literature for this species consists predominantly of Western blot, RT-PCR, and immunohistochemistry findings—i.e., expression changes correlated with treatment but not formally validated through knockdown, rescue, or genetic models. Mechanistic claims should therefore be read as associations rather than established mechanisms; pathway-level conclusions require formal validation that has not yet been performed in this literature.
- *Publication bias.* Negative and null findings in preclinical studies of traditional medicines are systematically under-reported. The positive findings summarised in Section 6 may therefore overestimate the true effect size and consistency of pharmacological activities.

### 7.2 Pharmacokinetic and route-of-administration limitations

Two related considerations affect the translational interpretation of the *R. parvifolius in vivo* pharmacology literature: the physicochemical properties of the candidate active metabolites, and the route of administration used in efficacy studies.

Ursolic acid, the most extensively investigated single metabolite, has high lipophilicity (LogP approximately 5.7) and low aqueous solubility, with reported oral bioavailability in rodents of approximately 1%–3%. This profile reflects limited gastrointestinal absorption, P-glycoprotein-mediated efflux, and first-pass hepatic metabolism. No pharmacokinetic data have been published for suavissimoside R1, nigaichigoside F1, coreanoside F1, sanguiin H-6, or any other *R. parvifolius* saponin in any species, despite these metabolites being the candidate active metabolites across the anti-tumour, neuroprotective, and anti-fatigue literature.

Most *in vivo* efficacy studies reviewed in Section 6 used intraperitoneal (i.p.) administration—including key anti-tumour studies (Cao 2021 melanoma; Xu 2018 K562 xenograft; Ge 2014 K562) — or intragastric (i.g.) administration at relatively high doses. Intraperitoneal administration bypasses gastrointestinal absorption and first-pass hepatic metabolism, producing systemic exposure that cannot be replicated by oral aqueous decoction, the route used in traditional preparation.

Three implications follow:

Efficacy reported in i.p. studies cannot be assumed to translate to oral therapeutic use; confirmation of efficacy following oral administration at clinically achievable exposures is required before clinical translation.

The absence of plasma exposure data prevents dose-response interpretation and prevents rational clinical dose selection.

The active circulating species after oral dosing remain uncertain. Saponin glycosides may undergo gut-microbiome hydrolysis to aglycones before absorption, but the early aglycone-versus-glycoside comparison by Chen et al. (2011) — where the intact suavissimoside R1 glycoside was active in dopaminergic neuroprotection and the aglycone was not—suggests that hydrolysis may inactivate rather than activate the parent metabolite, at least for this activity.

Three pharmacokinetic studies are therefore a precondition for clinically-oriented development: (a) oral pharmacokinetic characterisation of ursolic acid and key saponins with plasma concentration-time profiles and absolute bioavailability against i.v. reference; (b) tissue distribution in target organs (liver, brain, bone marrow, tumour xenograft); and (c) metabolite identification in plasma and urine. Until these are completed, the preclinical efficacy literature should be regarded as preliminary, requiring further validation before clinical translation.

A further preparation variable that has received minimal systematic investigation in this species is the contrast between fresh and dried root material. Traditional preparations exclusively use dried root, whereas isolated reports of fresh-root analyses (e.g., Do et al. (1988), who used fresh roots for (−)-epicatechin and sterol isolation) suggest that the metabolite profile of fresh material may differ from that of conventionally dried material. Drying-related changes in moisture content, enzymatic activity during post-harvest processing, and the relative abundance of thermolabile metabolites (including some glycosides and volatile metabolites) could meaningfully alter both the chemical profile and the pharmacological output of subsequent extracts. To our knowledge, no direct head-to-head comparison of chemically characterised fresh versus dried *R. parvifolius* root extracts has been published; this represents a defined research gap relevant to standardisation.

### 7.3 Pathway-associated changes versus experimentally validated mechanisms

A recurring feature of the *R. parvifolius* pharmacology literature is that pathway findings are typically reported as Western blot, qPCR, or IHC observations of expression or activity changes in one direction (↑ or ↓) following treatment. These observations document a pathway *association* with treatment, but do not on their own establish a *mechanism* of action. Experimentally validated mechanisms—for example, through knock-down, rescue, genetic models, or specific pharmacological antagonism—are rare in the literature reviewed here. Across all included studies, only the early aglycone-vs-glycoside comparison by Chen et al. (2011) approaches mechanism validation (by showing that the intact suavissimoside R1 glycoside, but not its alkaline-hydrolysed aglycone, retains dopaminergic protection). The 2024–2025 mechanistic studies (Cheng et al., 2025; Wang et al., 2025) strengthen the pathway evidence but do not yet include knock-down/rescue confirmation. Throughout this review, language is therefore calibrated to distinguish pathway-associated changes (the dominant evidence type) from experimentally validated mechanisms (rare); directional annotations in Figure 3 should be read as the former rather than the latter.

### 7.4 Formulation–activity mismatch (traditional vs. experimental extracts)

The traditional clinical preparation of *R. parvifolius* root is aqueous decoction (6–15 g/day per the Guangdong monograph (Guangdong Food and Drug Administration (compiled), 2019)). However, the great majority of the modern pharmacology literature uses TSRP (total saponins from *R. parvifolius*, obtained via ethanolic extraction followed by n-butanol partitioning and macroporous-resin enrichment), other alcohol-based fractions, or pure metabolites. The chemical composition of TSRP differs substantially from that of aqueous decoction—TSRP is enriched in lipophilic triterpenoid saponins, whereas aqueous decoction contains predominantly water-soluble metabolites (tannins, sugars, low-molecular-weight phenolics, soluble saponins). Pharmacological findings obtained with TSRP cannot therefore be directly translated to expectations for traditional aqueous decoction, and *vice versa*. This formulation–activity mismatch is a major limitation of the current evidence base for translational interpretation.

### 7.5 Study quality, replication, and publication bias

Several quality limitations affect the *in vivo* literature. Group sizes are typically modest (n = 6–12 per group) and *a priori* power calculations are rarely reported, limiting detection of modest effects and robust dose-response characterisation; for example, the key A375 melanoma xenograft study by Cao et al. (2021) used n = 10–12 per group at a single TSRP dose. For several domains—acute toxicity, dopaminergic-neuroprotection mechanism, and several *in vivo* tumour-inhibition studies—the evidence is concentrated within one or two research groups without independent replication. Many of the older Chinese-language primary studies also appeared in journals with limited international peer review and should be treated as hypothesis-generating until independently replicated, whereas the 2024–2025 studies in higher-impact journals (Lu et al., 2024; Jia et al., 2025; Cheng et al., 2025; Wang et al., 2025; Kim et al., 2025; Zhang et al., 2025) provide an emerging stronger baseline.

### 7.6 Limitations: summary

Taken together, the principal limitations are the preponderance of *in vitro* and rodent studies with only a single small clinical observation; absent pharmacokinetics for any candidate active metabolite; the dominance of pathway-associated over experimentally validated mechanistic evidence; the formulation–activity mismatch between traditional decoction and modern TSRP-based extracts; and limited toxicological characterisation (Section 9).

## 8 Extraction methodology, quality control, and standardisation

### 8.1 Extraction strategies in current use

Across the literature, four principal extraction strategies are in use, each yielding distinct phytochemical profiles:

TSRP (total saponin) preparation: 70% ethanol extraction → petroleum ether wash → n-butanol partition → D101 macroporous resin (typically 40%–50% ethanol elution) → preparative HPLC where required (Du and Rao, 2003; Zhang et al., 2016b). This purification protocol yields a fraction enriched in triterpenoid saponins but largely depleted of organic acids, flavonoid glycosides, and polysaccharides present in the whole root extract.

n-Butanol extract: without further resin purification, used in the hepatoprotection literature (Du et al., 2005).

Aqueous decoction (traditional): 1:10 water, heated 60 °C to boiling, reflux or simple boiling, evaporation under reduced pressure, lyophilisation; this protocol is closest to traditional use and was employed by Wang et al. (2025) in their 2025 ferroptosis-mechanism study and by Huang et al. (2023) in their multi-index water extraction optimisation study.

Volatile oil: optimised hydro-distillation of fresh leaves with reported yields around 0.36% w/w (Cai et al., 2012).

Crude polysaccharide: 10× water, 90 °C, 90 min, alcohol precipitation (Hu, 2011).

Dispersible tablet formulation: Mei et al. (2023) developed a *R. parvifolius* total-saponin dispersible tablet using central composite design—response surface methodology, optimising microcrystalline cellulose, crospovidone, and low-substituted hydroxypropyl cellulose composition; the resulting tablet showed disintegration time under 3 min and cumulative dissolution above 88% at 50 min, representing one of the few formulation-development studies in the *R. parvifolius* literature.

The pharmacological implications of these extraction differences are considerable. TSRP, the most pharmacologically investigated fraction, is chemically distant from traditional decoction; aqueous extracts retain a chemical profile closer to traditional use but are correspondingly less well characterised pharmacologically. The 2025 ferroptosis study (Wang et al., 2025) is among the few well-characterised *in vivo* studies of an aqueous-extract preparation.

### 8.2 Quality control: current state and critical gaps

#### 8.2.1 The 2019 Guangdong provincial standard monograph for máoméigēn

The principal pharmacopoeial reference for *R. parvifolius* in current Chinese practice is the monograph on “Máoméigēn” (*Rubi* parvifolii Radix) in the Guangdong Provincial Standard for Chinese Medicinal Materials (Third Volume, 2019, pp. 312–315) (Guangdong Food and Drug Administration (compiled), 2019). The monograph defines the drug as the dried root of *R. parvifolius* L (Rosaceae), collected from autumn to the following spring, free from rootlets and adhering soil, washed and dried. The standard was drafted by the Guangdong Institute for Drug Quality Research in conjunction with Kangmei Pharmaceutical Co. Ltd. and verified by the Guangdong Institute for Drug Control.

The 2019 monograph specifies:

Macroscopic description—cylindrical, often distorted root, 10–30 cm long and 0.3–1.2 cm in diameter, with grey-brown to brown surface, longitudinal wrinkles, occasional flaky exfoliation revealing a reddish-brown subsurface, hard texture, light-yellow radial-patterned section, slight odour, slightly astringent taste.

Powder-microscopic identification—characteristic features include surface-view polygonal/rectangular cork cells, thick-walled fibres in bundles (12–30 μm diameter), wood parenchyma with moniliform thickening, abundant calcium oxalate cluster crystals (20–40 μm), calcium oxalate prismatic crystals, secretory cells containing reddish-brown contents, and bordered-pit vessels.

Thin-layer chromatographic (TLC) identification — 2 g of powdered drug is extracted with 20 mL ethyl acetate under ultrasonication for 20 min; the filtrate is concentrated to 1 mL and applied alongside an authenticated *R*. *parvifolius* root reference drug onto a silica-gel G plate. Development uses n-hexane–ethyl acetate–formic acid (10:9:1); detection uses 5% vanillin–sulfuric acid spray followed by heating. Specification requires the test article to show spots of identical colour and Rf to the reference drug at the corresponding positions.

Numerical quality limits (compiled from the 2019 standard):

*Moisture*: ≤15.0% (Pharmacopoeia *of the People’s Republic of China* 2015; General Rule 0832, Method II); *Total ash*: ≤7.0% (General Rule 2,302); *Dilute-ethanol extractable matter* (hot-extraction method, General Rule 2,201): ≥12.0%; *Acid-insoluble ash*: not specified as a numerical limit (the standard reports measured values of 0.4%–0.5% across three test batches as too low to warrant a formal limit).

Reported chemical composition in the monograph: tannins, sugars, flavonoid glycosides, phenolics, amino acids, β-sitosterol, β-carotene, euscaphic acid, and suavissimoside R1, among others.

Functional indications (qīng rè jiě dú, qū fēng lì shī, huó xuè liáng xuè): pyrexia, sore throat, rheumatic pain, damp-heat jaundice, damp-heat diarrhoea, oedema, urinary lithiasis, haemoptysis, haematemesis, metrorrhagia, traumatic injury, carbuncles, mumps, scrofula, eczema, and pruritus. Dosage: 6–15 g (decoction). Contraindication: pregnancy.

#### 8.2.2 What the 2019 monograph does and does not provide

It is important to characterise this monograph accurately. The 2019 standard establishes (a) a formal botanical identification, (b) a powder-microscopic identification panel, (c) a single TLC fingerprint test against a reference drug (without a defined chemical marker), and (d) limits for moisture, total ash, and extractable matter. The monograph **does not** specify:

- a quantitative content limit for any single marker metabolite (e.g., ursolic acid, oleanolic acid, or any saponin); a multi-wavelength HPLC fingerprint; a reference-standard chemical marker for the TLC step (the comparison is against an authenticated reference *drug*, not against a pure reference *standard*).

The 2019 monograph therefore provides botanical and basic chemical identification but does not extend to multi-method chemical fingerprinting or quantitative marker-metabolite limits.

#### 8.2.3 Complementary quality-control research

Complementary to the 2019 monograph, Huang et al. (2016) developed and validated an HPLC method for L-epicatechin content determination across root, stem, and leaf; Lu et al. (2024) published the integrated four-tier botanical identification framework (macroscopic + microscopic + TLC + ITS rDNA) that strengthens the species-authentication component of any future updated standard; and (Jia et al., 2025) reported a UPLC-MS/MS plus network pharmacology framework that could form the basis of a future multi-metabolite quality-control specification. Together, these works define a feasible path toward an updated pharmacopoeial framework for *R. parvifolius* but are not themselves currently incorporated into the 2019 monograph.

#### 8.2.4 Quality-control gaps

The principal gaps in the current quality-control framework for *R. parvifolius* are:

The 2019 monograph does not include a quantitative marker assay, and no nationally recognised minimum content limit for any active metabolite has been published. Quantitative HPLC determination of triterpenoid markers (ursolic acid, oleanolic acid, suavissimoside R1, nigaichigoside F1) in *R. parvifolius* root would substantially strengthen the pharmacopoeial framework but has not been formally adopted.

Total saponin (TSRP) preparations used in pharmacological research show inter-laboratory and inter-batch variability in reported purity and composition (Section 7.4); no standardised reference TSRP preparation is commercially available for cross-laboratory comparison.

The species is not currently included in the *Pharmacopoeia of the People’s Republic of China* (2020 edition); pharmacopoeial coverage is limited to the regional Guangdong standard.

Inter-batch and inter-geographic-source quantitative chemical comparison studies have not been published. The 2025 chemotaxonomic GC-MS comparison across three Rubus species (Zhang et al., 2025) is a first step in this direction.

Closing these gaps—especially the introduction of a quantitative HPLC assay and inter-laboratory characterisation of TSRP composition—would substantially advance the regulatory and translational-research framework for *R. parvifolius* (see Section 11.7).

### 8.3 Preparation–activity mismatch: a critical consideration

A central and consistently underappreciated issue in this literature is the chemical mismatch between traditional preparation (aqueous decoction) and most experimental extracts (alcohol-based fractions and TSRP). Aqueous decoction is expected to extract predominantly saponin glycosides, flavonoid glycosides, polysaccharides, and organic acids, with limited extraction of lipophilic triterpenoid aglycones; alcohol-based and resin-purified preparations selectively enrich lipophilic triterpenoids at the expense of hydrophilic glycosides. The pharmacological relevance of experimental findings obtained with TSRP or n-butanol fractions to traditional oral decoction use therefore requires explicit caution. Going forward, ethnopharmacology-guided *R. parvifolius* research should prioritise aqueous-extract chemical profiling alongside lipophilic fractions, and the 2025 ferroptosis study (Wang et al., 2025) (which used an aqueous extract) and the integrated UPLC-MS/MS and network pharmacology study (Jia et al., 2025) provide useful methodological templates.

## 9 Toxicological profile and safety

### 9.1 Current acute and subacute evidence

Available toxicity data for *R. parvifolius* are limited to acute exposure characterisation. Wang et al. (2010) reported that TSRP up to 3.0 g/kg (intraperitoneal) and 10 g/kg (intragastric) in mice produced no observable acute toxicity, with LD50 not reached at the tested dose ceiling. Therapeutic doses in efficacy studies (5–100 mg/kg) are 31- to 1000-fold below these acute thresholds. Wang et al. (2025) noted no adverse events at the ERPL doses used in their 8-week chronic fibrosis study, although formal histopathological assessment of off-target organs was not the primary aim of that study.

The traditional safety record is generally favourable; *R. parvifolius* is consumed as an edible fruit (with smaller traditional culinary use) and the root has decades of regional ethnomedical use without documented serious adverse events. Pregnancy contraindication is noted in the Guangdong Provincial Standard monograph (Guangdong Food and Drug Administration (compiled), 2019) based on traditional experience.

### 9.2 Critical limitations of the available toxicology data

The toxicology evidence base has several substantial limitations that we now consider explicitly. First, all reported acute-toxicity data come from a single research group (Wang et al., 2010), and have not been independently replicated. Second, none of the doses tested correspond to chronic clinically relevant exposure: the LD50 ceiling experiments use single-dose acute exposure, which is poorly predictive of subchronic and chronic adverse effects. Third, the route used in acute-toxicity testing (intraperitoneal) bypasses gut absorption and first-pass hepatic metabolism—the routes most relevant to traditional oral use—and so cannot be used to derive oral safety margins directly. Fourth, the dose-range relevance to human exposure is not characterised: traditional oral dosing of *R. parvifolius* root is 6–15 g of crude drug per day (Guangdong Food and Drug Administration (compiled) (2019), and the corresponding systemic exposure to candidate active metabolites (suavissimoside R1, nigaichigoside F1, sanguiin H-6, ursolic acid) is unknown because no pharmacokinetic study has been published (Section 7.2; Section 9.3). Fifth, off-target organ histopathology in any published *R. parvifolius* toxicity study is limited; the Wang et al. (2025) chronic fibrosis study did not include detailed hepatic, renal, cardiac, or gonadal histopathological evaluation as a primary endpoint.

### 9.3 Regulatory gaps relevant to clinical translation

The following regulatory-grade gaps remain and are direct preconditions for any clinical development of *R. parvifolius* preparations:

No formal subchronic (28- or 90-day) toxicity study has been published for any *R. parvifolius* preparation.

No chronic toxicity study has been published.

No genotoxicity assessment (Ames, micronucleus, comet) has been published, despite the relevance of this assessment for any preparation considered for chronic clinical use.

No reproductive or developmental toxicity study has been published; the traditional pregnancy contraindication is empirical rather than evidence-based.

No formal pharmacokinetic study—and therefore no exposure-response framework—has been published for any candidate active metabolite of this species in any species (rodent or human).

No human pharmacovigilance **data** are formally documented; long-term traditional use in southern China provides some background reassurance but cannot substitute for systematic post-marketing surveillance in a clinical-pharmaceutical context.

### 9.4 Implications for risk assessment

Given the limitations above, the absence of reported adverse events in the existing literature should not be interpreted as evidence of safety. The current toxicology evidence base supports only the conclusion that *R. parvifolius* root preparations, at the acute exposure levels tested, do not produce overt toxicity in mice. It does not support any of the following: (i) safety for chronic human use; (ii) safety for use in pregnancy, lactation, or paediatric populations (even setting aside the traditional contraindication); (iii) safety in combination with prescription pharmaceuticals (no drug–drug interaction studies have been performed); or (iv) safety in patients with hepatic or renal impairment. These gaps are addressed as a priority research direction in Section 11.2.

## 10 Discussion

This synthesis integrates the published *R. parvifolius* literature (1988–May 2026) to provide an integrated English-language account of *R. parvifolius* phytochemistry and pharmacology that incorporates the 2024–2025 mechanistic literature. Four thematic considerations warrant focused discussion.

### 10.1 Breadth of pharmacological profile and the TSRP-Centred evidence base

The reported pharmacological profile of *R. parvifolius* spans multiple activity categories. TSRP shows reproducible activity across anti-tumour, neuroprotective, anti-fatigue, and anti-hepatic-fibrosis endpoints in independent research groups, making TSRP the most extensively investigated experimental fraction. Reported pathway associations involve apoptosis-related signalling (STAT3, Bcl-2/Bax), cellular energetics (AMPK), ferroptosis-related signalling in liver fibrosis (p53/SLC7A11/GPX4), and PI3K/AKT-related signalling in imatinib resistance, across multiple disease models. These findings indicate activity across multiple disease models, and align with the 2025 network pharmacology analysis by Jia et al. (Jia et al., 2025) identifying AKT1 as a principal target hub.

However, the breadth of activity must be qualified. With rare exceptions noted in Section 6, the mechanistic literature consists of expression-change associations rather than established mechanisms. Whether the convergent pathway associations reflect direct ursane saponin target engagement, indirect modulation through upstream signalling, or a combination of mechanisms requires formal validation through knockdown, rescue, genetic models, or chemical genetics. The multi-target hypothesis is plausible and consistent with the pharmacology of complex saponins generally, but the current evidence is best characterised as supporting the *hypothesis* of multi-target activity rather than as establishing specific mechanisms.

The 2024–2025 mechanistic literature has added substantially to the *R. parvifolius* evidence base in three directions. First, Cheng et al. (Cheng et al., 2025) reported TSRP activity in imatinib-resistant K562 (K562R) cells, with reversal of resistance associated with PI3K/AKT pathway downregulation; this is the first reported imatinib-resistance reversal study for this species and links it to a clinically relevant resistance mechanism in chronic myeloid leukaemia. Second, Wang et al. (Wang et al., 2025) reported the first ferroptosis-linked hepatic activity for *R. parvifolius*, with p53/SLC7A11/GPX4 expression changes in a CCl4-induced fibrosis model; this is the first study linking *R. parvifolius* to a regulated cell death modality other than apoptosis. Third, Kim et al. (Kim et al., 2025) provided the first quantitative leaf-flavonoid characterisation with dose-resolved anti-inflammatory activity in atopic dermatitis-relevant cell models, identifying quercetin 3,7-diglucoside (12.73 mg/g DW), hirsutrin (4.74 mg/g DW), and ellagic acid as the dominant leaf metabolites. Each of these reports requires confirmatory validation by independent groups, but together they signal an expansion of the *R. parvifolius* pharmacological repertoire beyond the historical TSRP-anti-tumour and TSRP-neuroprotection focus that dominated the 1994–2019 literature.

### 10.2 Traditional–pharmacological correspondence: a qualified assessment

The correspondence between traditional indications and modern preclinical findings for *R. parvifolius* is broad across most indication categories—haemostasis, hepatoprotection, anti-inflammatory activity, anti-tumour activity, neuroprotection, anti-fatigue, and bone-related indications all show preclinical findings consistent with traditional use. These findings align with several traditional indication categories. They do not, however, establish that traditional preparations achieve the same effects clinically.

A more accurate characterisation than ‘traditional uses are validated’ is: the preclinical pharmacological findings for R. parvifolius* align with the traditional ethnomedicinal indications, with the qualifications that (i) the preclinical evidence is almost entirely from cell-based and acute rodent models with limited predictive validity for clinical efficacy; (ii) publication bias likely contributes to the observed correspondence; (iii) traditional aqueous preparations differ chemically from most experimental extracts used to generate the supporting preclinical data; and (iv) mechanistic claims are associations rather than established mechanisms.* This characterisation provides a balanced foundation for further translational investigation without overstating the strength of the underlying evidence.

### 10.3 Brief note on research priorities

The 2024 four-tier botanical identification framework by Lu et al. (2024) — combining macroscopic, microscopic, TLC, and ITS rDNA molecular methods—provides the most comprehensive identification protocol available for this species. Adoption of this framework in any future *R. parvifolius* pharmacological study would substantially reduce the risk of botanical misidentification, particularly within the hollow-stemmed *Rubus* group where 53 related species share habitat and morphology. This represents the methodological infrastructure required for chemically reproducible *R. parvifolius* research going forward.

The five priority research gaps that emerge from this synthesis are presented in detail as an independent section below (Section 11: Priority Research Gaps and Future Directions) to make the translational research agenda explicit and easily locatable for readers.

### 10.4 Comparison with *rubus* congeners

*Rubus idaeus (raspberry)* and *Rubus occidentalis* (black raspberry) have been more extensively studied in cancer chemoprevention, primarily through fruit-derived anthocyanin-rich extracts; the chemical and pharmacological profiles of fruit-derived *Rubus* preparations differ from *R. parvifolius* root preparations (Park et al., 2017). R. chingii and *Rubus coreanus* share more chemical features with *R. parvifolius* (the ursane saponin profile) and have been independently characterised in lipid metabolism, antioxidant, and reproductive-tonic contexts. The high ursane triterpenoid saponin content of *R. parvifolius* root distinguishes it from these fruit-derived preparations and may contribute to differences in the reported pharmacological profiles of root and fruit-derived preparations. No direct head-to-head comparative pharmacological study has been published; the suggestion that root preparations may offer activity advantages for specific oncological or neuroprotective applications remains hypothesis-level and requires direct experimental comparison.

A 2025 GC-MS chemotaxonomic comparison of three *Rubus* species—R. rosifolius, *R. parvifolius*, and *R. idaeus*—by Zhang et al. (2025) further documented distinct volatile and lipophilic metabolite profiles among these species, supporting the view that pharmacological extrapolation across *Rubus* congeners requires explicit chemical characterisation of the preparation in question.

### 10.5 Implications for clinical practice and regulatory decisions

The current evidence base for *R. parvifolius* does not support specific clinical recommendations or regulatory endorsement of any preparation for any indication. The single published clinical study (Xu et al., 2012) is a small non-blinded single-arm observation with significant methodological limitations and should not inform clinical decisions. Traditional aqueous decoction has a centuries-long safety record consistent with continued use in established ethnomedical contexts under appropriate clinical supervision; however, the chemical and pharmacological discrepancy between traditional aqueous decoction and the modern experimental extracts (TSRP, n-butanol fractions) means that preclinical findings of the latter cannot be directly translated to clinical recommendations for the former. For regulatory consideration of any *R. parvifolius* preparation in any clinical indication, the priority requirements are: (i) IND-enabling toxicology; (ii) oral pharmacokinetic characterisation of the candidate active metabolites; and (iii) prospective clinical trials in defined indications with appropriate placebo or active-comparator control. Until these requirements are met, the evidence base supports continued preclinical investigation but not clinical translation.

## 11 Priority research gaps and future directions

Five priority research gaps emerge from the present synthesis, presented in approximate order of translational impact:

### 11.1 Pharmacokinetics (highest priority)

No oral pharmacokinetic study has been published for any *R. parvifolius* metabolite in any species. Establishing systemic exposure parameters for the principal metabolites—ursolic acid, suavissimoside R1, nigaichigoside F1, sanguiin H-6, and ellagic acid—is the single highest-impact translational research priority. Without exposure data, efficacy-to-dose relationships are uninterpretable, and the preclinical efficacy literature cannot inform clinical dosing rationally. Full discussion of this priority is provided in Section 7.2.

### 11.2 Formal toxicology

Formal subchronic (90-day), chronic, genotoxicity, reproductive, and developmental toxicity characterisation to IND-enabling standards is a precondition for clinical development of any *R. parvifolius* preparation. Acute toxicity data alone are insufficient. The absence of published toxicological characterisation must not be interpreted as evidence of safety.

### 11.3 Aqueous extract chemical profiling

The chemical profile of aqueous root decoction—the traditional preparation—has been characterised much less thoroughly than alcohol-based fractions. Systematic UPLC-MS/MS profiling of aqueous extract alongside parallel profiling of TSRP and other lipophilic preparations would address a key gap and would inform interpretation of which preclinical findings can reasonably be expected to translate to traditional clinical use. The 2025 Wang Guo aqueous-extract study (Wang et al., 2025) (anti-hepatic-fibrosis ferroptosis) is a useful methodological template for future aqueous-extract chemical-pharmacological correlation studies.

### 11.4 Fresh versus dried root comparison

Traditional preparations use dried root, but the metabolite profile of dried versus fresh material has not been systematically compared in this species. A focused comparative study—applying matched extraction conditions to fresh and conventionally dried root material with parallel UPLC-MS/MS and bioactivity profiling—would clarify whether drying-related compositional shifts have meaningful pharmacological consequences. Such a study would inform both standardisation choices and the interpretation of pharmacological findings reported from preparations of unspecified post-harvest history.

### 11.5 Triple-negative breast cancer as a hypothesis-generating direction

The cross-species data summarised in Section 6.1.3 — sanguiin H-6 cytotoxicity in MDA-MB-231 cells (a triple-negative breast cancer [TNBC] model) via an oestrogen receptor-independent mechanism, and anti-metastatic activity in the same cell line through VEGF/Akt/ERK1/2 signalling—provide indirect mechanistic rationale for prioritising TNBC over hormone receptor-positive subtypes when designing direct *R. parvifolius* breast cancer studies. TNBC is the breast cancer subtype with the greatest unmet therapeutic need, characterised by absence of endocrine and HER2-directed treatment options and by a more aggressive disease course. The convergence of (a) documented sanguiin H-6 presence in *R. parvifolius* root, (b) hormone receptor-independent activity of this metabolite in cross-species TNBC models, and (c) the multi-target preclinical profile of *R. parvifolius* triterpenoid fractions defines TNBC-specific cell-line and xenograft studies as a defensible research priority. Such studies should use chemically characterised *R. parvifolius* preparations of standardised composition (Section 11.4) and quantify sanguiin H-6 content alongside any reported activity, to permit cross-species mechanistic comparison.

### 11.6 Prospective clinical trial design

The current clinical evidence base is limited to a single small non-blinded single-arm observation (Xu et al., 2012). Even before formal randomised controlled trial design, a well-conducted prospective dose-finding and safety cohort in an appropriate indication—most plausibly imatinib-resistant chronic myeloid leukaemia, given the 2025 Cheng Jiajun mechanistic findings (Cheng et al., 2025) — would advance the clinical evidence base. Any such trial design must explicitly account for the pharmacokinetic and route-of-administration considerations discussed in Section 7.2.

### 11.7 Updated pharmacopoeial quality standards

Updated pharmacopoeial quality standards incorporating: (a) the 2024 four-tier botanical identification framework of Lu et al. (2024) (macroscopic + microscopic + TLC + ITS rDNA); and (b) the 2025 integrated UPLC-MS/MS plus network-pharmacology methodology of Jia et al. (2025) (multi-metabolite quality control with mechanistically informed marker selection) — would consistently strengthen the regulatory and quality-control framework for *R. parvifolius* preparations.

### 11.8 Limitations of this review

This review itself has the following acknowledged limitations: (i) literature coverage is limited to publications through May 2026; (ii) no formal risk-of-bias assessment was performed (consistent with the narrative-review approach); (iii) no meta-analysis was performed, given substantial heterogeneity in extract type, model, and dosing across the included studies; (iv) the majority of primary studies are published in Chinese, which may constrain accessibility for some readers; (v) directional annotations (↑/↓) in Figure 3 reflect reported expression or activity changes rather than experimentally validated mechanisms (Section 7); (vi) a subset of the primary pharmacological studies summarised here use single-dose comparison designs rather than full dose-response characterisations, and the conclusions of such studies should be interpreted within those constraints—dose-response validation remains required for translational development; (vii) the single available clinical observation (Xu et al., 2012) is reported with explicit acknowledgement of its non-blinded, non-randomised single-arm design and cannot establish clinical efficacy or safety.

## 12 Conclusion

*Rubus parvifolius* L. is a pharmacologically diverse Asian medicinal plant with an accumulating preclinical literature anchored by ursane- and oleanane-type triterpenoid saponins (TSRP), polyphenolic ellagitannins (sanguiin H-6), flavonoid glycosides, and phenolic acids. TSRP is the most extensively investigated fraction. Preclinical activity has been documented across anti-tumour (melanoma and chronic myeloid leukaemia), neuroprotective (cerebral ischaemia and Parkinson’s disease models), hepatoprotective (acute injury and 2025 evidence for ferroptotic mechanism in liver fibrosis), antioxidant, anti-inflammatory (with leaf > root > stem activity ordering), antibacterial (leaf volatile oil), anti-fatigue (with nigaichigoside F1 as the candidate active metabolite), and anti-osteoclastogenic categories. The 2025 UPLC-MS/MS plus network pharmacology characterisation by Jia et al. and the 2025 PI3K/AKT mechanistic finding by Cheng Jiajun et al. in imatinib resistance reversal extend the mechanistic literature.

Preclinical findings align with traditional ethnomedicinal indications across multiple categories. The principal translational gaps—absence of pharmacokinetic characterisation for the saponin metabolites, predominance of intraperitoneal routes in preclinical efficacy studies, and limited human evidence—are detailed in Sections 7, 11. Addressing these gaps will determine whether *R. parvifolius* preparations can transition from a preclinically promising regional folk medicine to a clinically validated phytotherapeutic.
